# The effects of blood flow restriction training on post activation potentiation and fatigue level: systematic review with meta-analysis

**DOI:** 10.3389/fphys.2025.1558008

**Published:** 2025-04-10

**Authors:** Nannan Zeng, Haiyang Liu, Jian Wang, Lizhu Jiang

**Affiliations:** ^1^ School of TCM and Pharmacology Health and Early Childhood Care, Ningbo College of Health Sciences, Ningbo, China; ^2^ Department of Physical Education, Ningbo University of Technology, Ningbo, China; ^3^ College of Education, Ningde Vocational and Technical College, Ningde, China

**Keywords:** blood flow restriction training, rate of perceived exertion, blood lactate, fatigue level, metaanalysis

## Abstract

**Objective:**

This study aims to comprehensively explore the effects of blood flow restriction (BFR) training on the post-activation potentiation (PAP) and the rating of perceived exertion (RPE) through meta-analysis, so as to provide a scientific basis for athletes’ physical fitness training.

**Methods:**

The PubMed, CNKI, Web of Science and EBSCO databases were searched to look for randomized controlled trials (RCTs) on the effects of BFRT on post-activation potentiation (PAP) and the rating of perceived exertion (RPE). The Cochrane risk of bias tool was used to screen the literature. With the help of Revman 5.4 and Stata 17.0 software, the literature quality assessment and statistical analysis were completed. Meanwhile, sensitivity analysis and funnel plots were utilized to examine the stability of the results and the publication bias.

**Results:**

A total of 31 related studies were included, among which 22 studies focused on the relationship between BFR training and PAP, covering 347 participants. Meta-analysis showed that BFRT could significantly affect PAP [*SMD* = 0.49, 95% CI (0.20, 0.77), *P* = 0.0008]. When the testing method was Squat Jump [*SMD* = 1.35, 95% CI (0.40, 2.30), *P* < 0.0005], the exercise intensity was 40%–70% 1RM [*SMD* = 1.21, 95% CI (0.69, 1.73), *P* < 0.0001], and the compression intensity was ≤50% AOP [*SMD* = 0.77, 95% CI (0.24, 1.30), *P* = 0.05], the effects on PAP reached the maximum, respectively, and were statistically significant. In terms of the impact of BFR training on RPE, 18 studies with 238 subjects were included. BFR training could significantly increase the RPE of the subjects [*SMD* = 1.21, 95% CI (0.69, 1.73), *P* < 0.0001]. When the exercise mode was Knee flexion [*SMD* = 0.65, 95% CI (0.18, 1.11), *P* = 0.0006], the exercise intensity was Mixed oxygen Training [*SMD* = 1.03, 95% CI (0.50, 1.56), *P* = 0.0001], and the compressive strength was ≥60% AOP [*SMD* = 0.75, 95% CI (0.02, 1.48), *P* = 0.05], a more significant effect size was presented.

**Conclusion:**

Blood flow restriction training can induce the occurrence of post-activation potentiation (PAP). BFR exercises with 40%–70% 1RM and ≤50% AOP are more likely to stimulate PAP. Meanwhile, BFR training will significantly affect the rating of perceived exertion (RPE). BFR training under mixed oxygen training and with a compressive strength of ≥60% AOP has a stronger perception of fatigue.

**Systematic Review Registration:** http://inplasy.com, identifier INPLASY202430008.

## 1 Introduction

In the domains of sports training and rehabilitation, the ceaseless exploration and innovation of methodologies to augment training efficacy, optimize athletic performance, and judiciously modulate the body’s fatigue response have perpetually constituted research hotspots. Conventional training protocols typically utilize high-intensity loads; however, prolonged high-intensity training cycles exceeding the musculoskeletal tolerance threshold may elevate injury risk and neuromuscular fatigue (Al Attar et al., 2022). This is especially pronounced for patients during the rehabilitation phase, the elderly cohort, or those with subpar physical fitness, where the implementation of high-intensity training is circumscribed (Fahlström et al., 2002). In contrast, blood flow restriction (BFR) training training has pioneered novel pathways. By strategically applying suitable pressure to the proximal segments of the limbs, thereby partially impeding blood circulation, it empowers muscles to activate a series of intricate physiological adaptation mechanisms even under relatively low-intensity exercise conditions (Yasuda et al., 2015). Initially, this training philosophy was tentatively employed within a narrow scope in the realm of rehabilitation medicine, with the express aim of expediting the recovery process for patients incapacitated by injuries or ailments from enduring traditional high-intensity training (Behrendt et al., 2020). As time elapsed and relevant research amassed, BFR training progressively transcended the boundaries of rehabilitation, permeating into arenas such as competitive sports and fitness training (Hanke et al., 2020).

Post-Activation Potentiation (PAP), a captivating physiological phenomenon, manifests as a transient enhancement in subsequent athletic performance following a high-intensity muscular stimulation (Tillin and Bishop, 2009). Concurrently, the Rate of Perceived Exertion (RPE) bears an intimate nexus with the training experience and compliance of trainees (Arney et al., 2019). The investigational timeline of BFR in PAP and fatigue research traces to Loenneke’s 2010 hypothesis on ischemic PAP potentiation (Loenneke et al., 2011). Following early advancements, systematic BFR research expanded exponentially, with landmark randomized controlled trials (RCTs) documenting PAP persistence for 15–30 min following intervention protocols (Xenofondos et al., 2015). Conversely, RPE studies diverged significantly–early rehabilitation trials consistently reported reduced perceived exertion, whereas later sports science applications paradoxically observed elevated RPE under occlusion (Serra et al., 2024).

While prior systematic reviews have extensively documented BFR’s effects on muscle hypertrophy and strength recovery in clinical populations (Liu et al., 2024), these analyses predominantly focus on chronic adaptations over weeks or months of training. Recent studies have demonstrated that BFR is an effective warm-up modality for enhancing explosive power (Wang et al., 2023). Thus, this systematic review and meta-analysis was conducted to evaluate the effects of BFR training on transient PAP and RPE. The overarching aspiration is to proffer robust scientific scaffolding for sports training, rehabilitation practices, and the expansion of germane theories, along with furnishing strategies for the precise and efficacious application of BFR training.

## 2 Materials and methods

### 2.1 Search strategy

In accordance with the PRISMA statement (Moher et al., 2009), two independent investigators carried out double-blind literature searches on PubMed, Web of Science, EBSCO, and CNKI databases. The search period spanned from the inception of each database until 24 December 2024, and a total of 31 articles were ultimately incorporated. Boolean logic was systematically applied to combine search terms across three domains: (“blood flow restriction training” OR “KAATSU” OR “BFR” OR “pressure training” OR “occlusion training”) AND (“strength” OR “PAP” OR “post activation potentiation” OR “upper body” OR “lower body” OR “fatigue” OR “RPE”) AND (“RCT” OR “randomized controlled trial”).

### 2.2 Inclusion and exclusion criteria

#### 2.2.1 Inclusion criteria

The inclusion criteria for literature were developed based on the PICOST principle (Li et al., 2014), with the following components explicitly specified:

Study Design: Published RCTs evaluating the effects of BFR training on upper limb muscle strength and fatigue (Include languages in Chinese and English). Participants: Healthy individuals (≤35 years), with no age restrictions. Intervention: BFR training implemented through various modalities (e.g., resistance exercise, aerobic training) with specific pressure parameters. Comparator: Alternative training methods without blood flow restriction (e.g., traditional resistance training, conventional aerobic exercise) or non-training control groups. Outcomes: Muscle strength (e.g., 1RM testing), Power output (e.g., jump height), Endurance capacity (e.g., Rate of Perceived Exertion), Functional performance (e.g., sprint times). Timeline: Immediate data after each group’s exercise is completed.

#### 2.2.2 Exclusion criteria

Non-RCT Designs: Non-randomized trials, observational studies, reviews, or case reports. Irrelevant Interventions: Studies not involving BFR training or focusing on non-upper limb interventions. Population Exclusions: Studies involving patients with chronic diseases or animal models. Outcome Exclusions: Studies lacking quantitative data on muscle strength or fatigue outcomes. Duplicate Publications: Redundant studies with overlapping datasets. Special Handling of Inaccessible Data: For articles where the full text is unavailable or the information is incomplete, the corresponding author will be contacted via email to request missing materials (Classified unresponsive studies as “Pending Analysis”).

### 2.3 Data extraction

**FIGURE 1:**
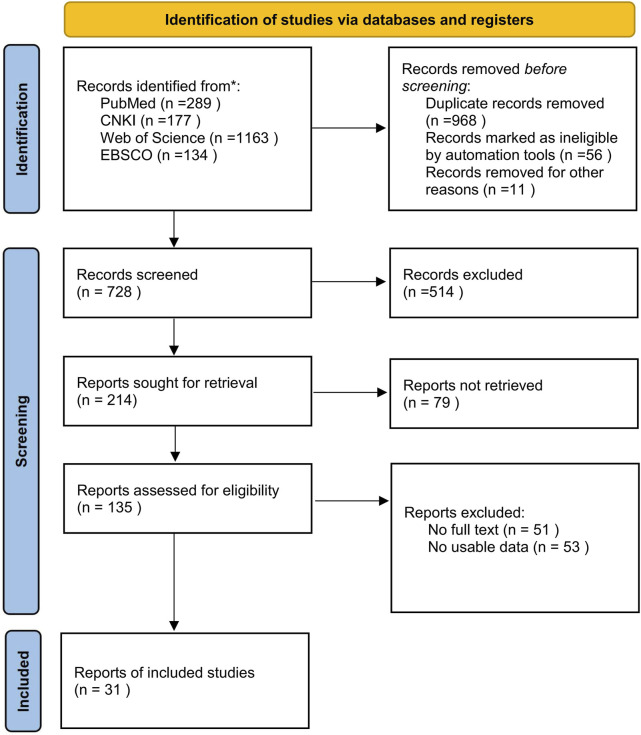
Flow diagram of literature selection.

The retrieved literature was uniformly imported into NoteExpress 3.9 software and screened independently by two researchers. The detailed steps of literature screening and inclusion are illustrated in Prisma flow diagram (Figure 1). After a rigorous selection process, two researchers extracted relevant information using a self-designed form, which encompassed the following aspects:

1. General Information: This included details such as the first author and the publication year.
2. Sample Information: Comprising the research subjects, their age, and the sample sizes of both the experimental and control groups.
3. Characteristics of Exercise Intervention: The intervention plan for the experimental group, which involved details such as training methods, training volume, and pressure intensity. These factors were critical in evaluating the nature and intensity of the applied interventions.

Outcome Indicators: Primarily focusing on quantitative indicators of PAP (Post-Activation Potentiation) and RPE (Rate of Perceived Exertion).

### 2.4 Statistical analysis

Statistical analyses were implemented using Review Manager 5.3. The outcome variables documented in the extant literature were continuous in nature. Considering the disparate testing methodologies employed for each indicator, standardized mean deviation (*SMD*) and 95% confidence interval were designated as the effect measurement metrics. Subgroup analyses for PAP outcomes were stratified by testing methods, exercise intensity, and compressive strength, while RPE outcomes were subgrouped by exercise mode, exercise intensity, and compressive strength. Risk of bias was evaluated using the Cochrane tool across seven domains: random sequence generation, allocation concealment, participant blinding, outcome assessor blinding, incomplete outcome data, selective reporting, and other biases. Heterogeneity was examined via the Heterogeneity test (*Q*-test), with a predetermined significance level of 0.1. The *I* ^2^ values fluctuated within the range of 0% to 100%, and when *I* ^2^ surpassed 50% and the p-value was less than 0.1, it signified the presence of significant heterogeneity, necessitating the selection of a random effects model for meta-analysis; otherwise, a fixed effects model was utilized. Moreover, Stata 17.0 was harnessed to perform sensitivity analysis, aiming to validate the stability of the resultant findings. The Egger test was executed and a funnel plot was constructed to explore potential publication biases.

## 3 Results

### 3.1 Study characteristics

A total of 31 randomized controlled trials (RCTs) were incorporated into this meta-analysis. The sample consisted of 459 participants. The age range of the participants was from 15 to 35 years old. The fundamental characteristics of the included literature are comprehensively presented in Table 1.

**TABLE 1:** Characteristic of studies included in systematic review and meta-analysis.

Study	Country	Age (years)	N (EG/CG)	Intervention (EG/CG)	Plan (BFR intensity)	Outcome extracted
Rivera et al. (2023)	United States	21 ± 2	14/14	BFR/No BFR	Four sets of 30–15–15–15 times 30% 1RM leg extensions (60% AOP)	RPE↑
Santiago-Pescador et al. (2022)	Spain	24.8 ± 7.0	15/15	BFR/No BFR	25 min knee flexion (50% AOP)	CMJ↓ RPE↑
Bielitzki et al. (2024a)	Germany	23.0 ± 2.9	15/15	BFR/No BFR	Four sets (30–15–15–15 repetitions) of knee extensions at 20% 1RM (60% AOP)	RPE↑
Zheng et al. (2022)	China	22.7 ± 2	12/12	BFR/No BFR	a set of five leg press repetitions, with a 60 s interval between the repetitions (50% AOP)	CMJ↑
Wernbom et al. (2009)	Sweden	25 ± 5	11/11	BFR/No BFR	Three sets of 30% 1RM unilateral knee extensions to failure (40% AOP)	RPE NS
Girard et al. (2019)	Germany	22.6 ± 1.3	14/14	BFR/No BFR	Four sets to failure at 70% 1RM biceps curl (60% AOP)	BC↑ PRE↑
Hornikel et al. (2023)	United States	24.8 ± 4.7	13/13	BFR/No BFR	Four sets of 75% 1RM squat four sets to failure (80% AOP)	CMJ↓ RPE↑
Victor et al. (2024)	Brazil	25.6 ± 3.78	10/10	BFR/No BFR	Keep increasing the speed of the treadmill until exhaustion (40% AOP)	PRE↑
Bielitzki et al. (2024b)	Germany	23.8 ± 3.1	24/24	BFR/No BFR	Static balance exercise on a BOSU ball (three sets of 60 s with 30 s rest in-between) on three separate (40% AOP)	SJ↑ PRE↑
Doma et al. (2020)	Australia	22.9 ± 5.0	18/18	BFR/No BFR	Three sets of eight reps each for single-leg lunges, leg swings, high knees and hip kicks (130 mmHg)	VJ NS
Hongwen and Xiang (2022)	China	23.6 ± 1.51	27/27	BFR/No BFR	Two sets of 10 straight leg jumps, followed by three sets of five consecutive obstacle jumps, and then do five drop jumps. (160 mmHg)	CMJ↑
Zheng et al. (2024)	China	21 ± 2	16/16	BFR/No BFR	Four groups 15 times 30% 1RM sitting squat (80% AOP)	VJ↑
Miller et al. (2018)	United States	21.8 ± 2.6	20/20	BFR/No BFR	Three sets of 20 s WVB Three sets of MVC (160 mmHg)	CMJ↑
Yan and Guo (2018)	China	20.3 ± 2.3	20/20	BFR/No BFR	Five sets of 50% HRR 2-min interval runs (150 mmHg)	CMJ↑
Zhang et al. (2023)	China	22.93 ± 0.78	16/16	BFR/No BFR	Five sets of isometric squat exercises with vibration plate (268.33 ± 20.83 mmHg)	20 m sprint↓
Cleary and Cook (2020)	United States	20.3 ± 0.9	15/15	BFR/No BFR	One set of 30 times 30% 1RM squats (60% AOP)	VJ NS
Sun and Yang (2023)	China	18.34 ± 1.88	12/12	BFR/No BFR	Four sets of 30–15–15–15 times 30% 1RM semi-squat (50% AOP)	CMJ↑ SJ↑ RPE NS
Hu (2020)	China	18.3 ± 3.28	12/12	BFR/HRT, LRT	Two groups of 30 + 20 30% 1RM squats (bundled pressure 40 mmHg, inflation pressure 200 mmHg)	CMJ↑
Schwiete et al., (2021)	Germany	22.8 ± 1.8	9/10	BFR/No BFR	Four sets of 30–15–15–15 times 20% 1RM leg press (183.3 ± 10 mmHg)	RPE↑
Zhang (2023)	China	20.20 ± 0.92	10/10	BFR/No BFR	Four sets of eight repetitions of bench press at 30% 1RM (140 mmHg)	BP↑ RPE↑
Husmann et al. (2017)	Germany	25 ± 4	17/17	BFR/No BFR	Four sets of 30-15-15-15 times 30% 1RM knee extension exercise (208 ± 25 mmHg)	RPE↑
Cornejo-Daza et al. (2024)	Spain	24.5 ± 4.8	20/20	BFR/No BFR	Three 组 8 次 60% 1RM full-squat (50% AOP)	CMJ↑
Serrano-Ramon et al. (2023)	Spain	23.6 ± 4.1	14/14	BFR/No BFR	Three repetitions of bench press at 60% 1RM (80% AOP)	BP↑
Duarte de Oliveira et al. (2023)	Brazil	27.1 ± 5.0	16/16	BFR/No BFR	Four sets of 30–15-15–15 times 20% 1RM bench press (150 mmHg)	RPE↑
Lei (2023)	China	23.67 ± 1.73	10/10	BFR/No BFR	Three sets of eight repetitions of bench press at 70% 1RM (180 mmHg)	RPE↑
Leszczynski et al. (2024)	Australia	18–35	20/20	BFR/No BFR	Three sets of 5 min knee extension exercise (80% AOP)	CMJ↑
Neto et al. (2017)	Brazil	18 ± 0.82	10/10	BFR/No BFR	Four sets of 30–15-15–15 times 20% 1RM bench press (163.80 ± 10.52 mmHg)	RPE NS BP NS
Che et al. (2022)	China	23.6 ± 3.1	10/10	BFR/No BFR	Four groups of 30–15-15–15 times 30% 1RM bench press (160 mmHg)	RPE↑
Wilk et al. (2020)	Poland	23.2 ± 2.66	12/12	BFR/No BFR	One repetition of bench press at 1RM (100% AOP, 135 ± 16 mmHg)	BP↑
Salagas et al. (2022)	Greece	25.8 ± 6	12/12	BFR/No BFR	Four sets of 12-s rapid bench press at 60% 1RM (100% AOP, 146 ± 15 mmHg)	RPE↓
Rodrigues et al. (2023)	Brazil	29.9 ± 5.9	15/15	BFR/No BFR	One repetition of bench press at 1RM (170 mmHg)	BP↑ RPE↓

NS, no statistical significance; ↑ represents a significant increase; ↓ represents a significant decrease; BP, maximum strength of bench press; RPE, rate of perceived exertion; SJ, squat jump; CMJ, counter movement jump; VJ, vertical jump; AOP, arterial occlusion pressure.

### 3.2 Study quality assessment

**FIGURE 2:**
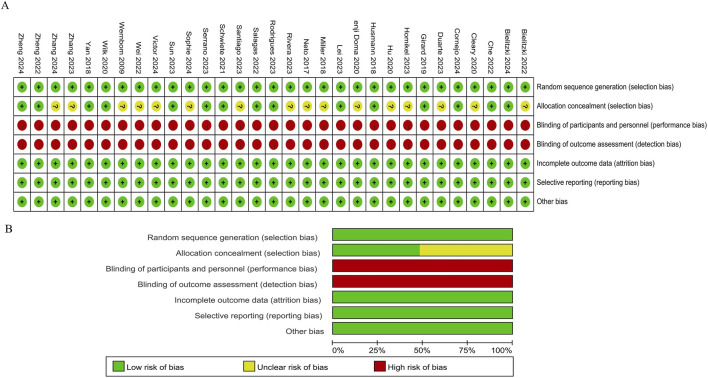
Methodological quality graph and summary of the included studies: **(A)** Risk of bias summary; **(B)** Risk of bias graph.

The literature quality was evaluated by employing the Cochrane risk bias assessment tool (Higgins and Altman, 2007). Among the included studies, 16 articles did not clarify whether random allocation was carried out. Moreover, all articles exhibited a high risk of blinding bias, which be attributed to the requirement of signing informed consent forms (Figures 2 A, B).

### 3.3 Post activation potentiation

**FIGURE 3:**
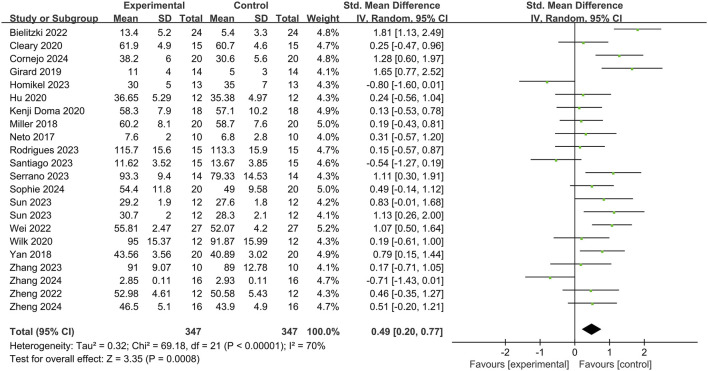
The effect of BFRT on PAP.

Among the 31 studies incorporated into this meta-analysis, 22 articles focused on exploring the relationship between Blood Flow Restriction (BFR) training and Post Activation Potential (PAP). The total number of participants involved was 347 (Figure 3). Heterogeneity testing was conducted, and the results revealed that *I* ^2^ = 70% > 50% and the *Q* test *P* < 0.01, suggesting a high degree of heterogeneity among the studies. Consequently, a random effects model was adopted for the meta-analysis. The findings indicated that the combined effect size, as measured by the Standardized Mean Difference (*SMD*), was 0.49, with a 95% Confidence Interval (*CI*) ranging from 0.20 to 0.77. The *Z* value was 3.13, and *p* = 0.0008 < 0.01, thereby demonstrating a significant correlation between BFR training and PAP.

### 3.4 Rating of perceived exertion

**FIGURE 4:**
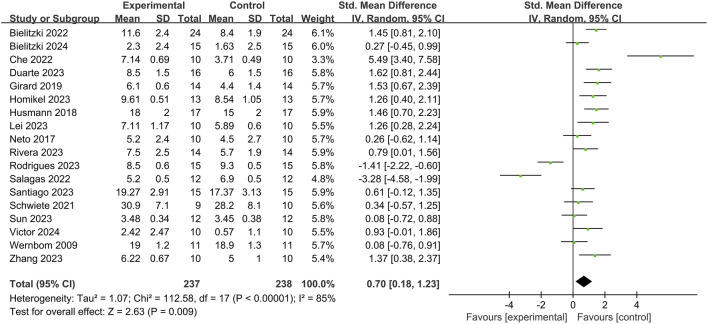
The effect of BFRT on RPE.

A total of 18 studies examined the impact of BFR on the Rating of Perceived Exertion RPE, involving 237 participants (Figure 4). Heterogeneity was high (*I* ^2^ = 85% > 50%, Q test *p* < 0.01), leading to the use of a random effects model for meta-analysis. Results showed that BFR training significantly improved RPE [*SMD* = 0.70, 95% *CI* (0.18, 1.23), *p* = 0.009].

### 3.5 Subgroup analysis

Significant heterogeneity (*I* ^2^ = 70%) was observed in pooled effects of BFR training on PAP. Subgroup analyses identified three primary sources: testing methods, exercise intensity, and compressive strength (Table 2).

**TABLE 2:** Subgroup analysis of BFR training on PAP and RPE.

Subgroup	Research features	Subgroup standard	Study (sample)	*SMD*	95% *CI*	*P*	*I* ^2^ (%)
PAP	Testing methods	Bench press	5 (61)	0.38	0.02, 0.75	0.41	0
		CMJ	10 (171)	0.44	0.03, 0.86	0.03	71
		Vertical Jump	3 (49)	0.29	−0.11, 0.68	0.16	0
		Squat Jump	2 (36)	1.35	0.40, 2.30	0.005	68
		Biceps Curl	1 (14)	1.65	0.77, 2.522	0.0002	N
		20 m sprint	1 (16)	−0.71	−1.43, 0.01	0.05	N
	Exercise intensity	≤30% 1RM	7 (87)	0.48	0.17, 0.78	0.002	0
		40%∼70% 1RM	2 (34)	1.21	0.69, 1.73	<0.0001	0
		One rep at maximum	2 (27)	0.17	−0.37, 0.70	0.54	0
		Exhaustive exercise	2 (27)	0.42	−1.98, 2.81	0.73	94
		Combined intensity	9 (172)	0.42	−0.08, 0.92	0.10	80
	Compressive strength	≤50% AOP	9 (137)	0.77	0.24, 1.30	0.005	76
		≥60% AOP	13 (210)	0.31	0.00, 0.62	0.05	58
RPE	Exercise mode	Bench press	7 (83)	0.65	−0.82, 2.12	0.39	93
		Knee flexion	5 (72)	0.65	0.18, 1.11	0.006	46
		Running	1 (10)	0.93	−0.01, 1.86	0.05	N
		Biceps Curl	1 (14)	1.53	0.67, 2.39	0.0005	N
		Squat	3 (35)	0.55	−0.16, 1.26	0.13	52
		BOSU ball	1 (24)	1.45	0.81, 2.10	<0.0001	N
	Exercise intensity	Mixed oxygen Training	3 (49)	1.03	0.50, 1.56	0.0001	32
		≤30% 1RM	10 (124)	0.93	0.35, 1.50	0.002	76
		40%∼70% 1RM	4 (49)	0.24	−1.59, 2.08	0.79	93
		One rep at Maximum	1 (15)	−1.41	−2.22, −0.60	0.0007	N
	Compressive strength	≤50% AOP	5 (72)	0.65	0.10, 1.21	0.02	60
		≥60% AOP	13 (165)	0.75	0.02, 1.48	0.05	88

N, no applicable.

Testing Methods: CMJ testing demonstrated moderate heterogeneity (*I* ^2^ = 71%), contrasting with homogeneous results for bench press and vertical jump (*I* ^2^ = 0%). Squat jump yielded the largest effect size (*SMD* = 1.35, *p* < 0.01), indicating BFR significantly improved jump height.

Exercise Intensity: Exhaustive/combined intensity protocols showed high heterogeneity (*I* ^2^ = 94% and 80%, respectively). Optimal effects occurred at 40%–70% 1RM (*SMD* = 1.21, *p* < 0.0001), with homogeneous results (*I* ^2^ = 32%).

Compressive Strength: Following Loenneke et al. (2015) classification of compressive intensity based on thigh circumference occlusion, subgroups were defined as ≤50% AOP (*I* ^2^ = 78%) and ≥60% AOP (*I* ^2^ = 58%). The ≤50% AOP subgroup achieved the highest effect size (*SMD* = 0.77, *p* = 0.005).

RPE Outcomes: Significant heterogeneity (*I* ^2^ = 82%) was observed. Knee flexion protocols showed the largest effect (*SMD* = 0.65, p = 0.006), while bench press demonstrated extreme heterogeneity (*I* ^2^ = 93%). Compressive intensities ≥60% AOP produced greater RPE effects (*SMD* = 0.75, *p* = 0.05).

### 3.6 Sensitivity analysis

To evaluate the robustness of findings, leave-one-out sensitivity analysis was conducted by sequentially removing individual studies from each subgroup (PAP and RPE). This iterative process aimed to identify studies contributing to heterogeneity and assess their impact on pooled estimates.

PAP outcomes: Initial pooled effect: *SMD* = 0.49 (95% *CI*: 0.20 to 0.77, *p* = 0.0008, *I* ^2^ = 70%). Removing any single study produced stable effects (*SMD*: 0.42 to 0.55, all *p* < 0.01), confirming no single study exerted disproportionate influence.

RPE outcomes: Excluding individual studies yielded consistent effects (*SMD* range: 0.65 to 0.88, all *p* < 0.05). Removal of Che (2022), Ridrigues (2023), and Salagas (2022) reduced subgroup heterogeneity (*I* ^2^ = 89%–79%), though overall heterogeneity remained substantial (*I* ^2^ = 85%).

These results highlight the meta-analysis’ stability despite moderate-to-high heterogeneity.

### 3.7 Publication bias

**FIGURE 5:**
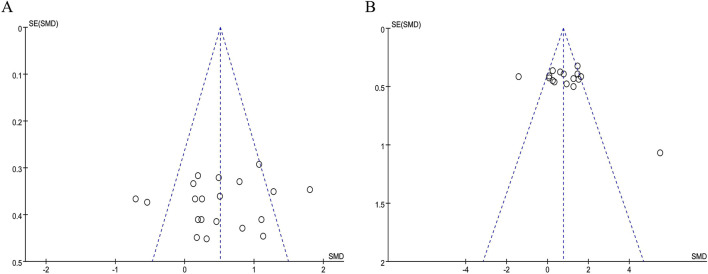
Funnel plot: **(A)** PAP; **(B)** RPE.

The publication bias of the included literatures was examined by drawing funnel plots. In Figure 5, the funnel plots were basically symmetrical, and all the p-values in the Egger test were greater than 0.05. Therefore, within the scope of the current study, it can be determined that there was no obvious publication bias in the studies on the impact of BFR training on PAP and RPE. This further indicates that the results of this study have relatively high credibility and reliability.

## 4 Discussion

### 4.1 Impact of BFR training on PAP

This meta-analysis aimed to explore the association between BFR training and the PAP. Among the 31 studies, 22 focused on the relationship between BFR training and PAP, involving a total of 347 participants. The results strongly confirmed that there is a significant correlation between BFR training and PAP, which means that BFR training is expected to become an effective means to enhance PAP. In physical fitness training, the rational application of BFR training can help optimize the training effect and improve athletic performance (Patterson and Ferguson, 2010). Due to the partial restriction of limb blood circulation, the metabolites (such as hydrogen ions, inorganic phosphates, etc.) produced by muscle contractions cannot be removed as quickly as in the state of normal blood circulation. The increase in the concentration of hydrogen ions will reduce the pH value within the muscles, which may change the conformation of muscle contractile proteins (Coyle et al., 1982), increase their sensitivity to calcium ions, and thus promote muscle contractions and trigger PAP (Dankel et al., 2016). In addition, after being stimulated, muscles will activate a series of intracellular signaling pathways, such as the mammalian target of rapamycin (mTOR) signaling pathway (Drummond et al., 2009). The activation of this pathway can promote the synthesis of ribosomal proteins, and then increase the synthesis of muscle proteins, leading to muscle hypertrophy and enhancing the ability to generate muscle strength, ultimately inducing PAP (Ozaki et al., 2014). Li et al. (2021) found in a study on a small sample of athletes that a specific pattern of BFR training could enhance the post-activation potential of muscles in the short term, and its effect echoed some of the findings in this meta-analysis, indicating that the promoting effect of BFR training on PAP has a certain degree of reproducibility.

However, the current studies showed a high degree of heterogeneity, which indicated that the included studies differed in many aspects, such as the specific protocols of BFR training (including pressure settings, training duration, and rest intervals), the individual characteristics of the participants (physical fitness, sports experience, etc.), as well as the measurement methods and timings of PAP. Therefore, subgroup analyses were conducted in this study according to the characteristics that might cause heterogeneity.

#### 4.1.1 Testing methods

Squat Jump (SJ) exhibited the largest PAP effect (*SMD* = 1.35, *p* < 0.01), likely due to BFR-induced metabolic stress favoring type II fiber recruitment or enhanced neuromuscular efficiency (Jbc et al., 2017; Wilk et al., 2021). In contrast, Countermovement Jump (CMJ) outcomes displayed substantial heterogeneity, suggesting inconsistencies in movement standardization, force-generation techniques, or equipment calibration across studies. While SJ protocols have been extensively studied, future research should prioritize standardizing CMJ testing conditions (e.g., countermovement depth, arm swing protocols) to reduce variability and clarify BFR’s role in dynamic performance metrics.

#### 4.1.2 Exercise intensity

When the exercise intensity is precisely controlled within the range of 40%–70% of the one-repetition maximum (1RM), the PAP effect reaches its highest level. Studies have found that BFR training with an intensity of 40%–70% 1RM not only enables athletes to achieve a significant increase in muscle strength in the short term but also prolongs the duration of the post-activation potentiation effect, and the synergistic improvement effect on muscle explosive power and endurance is remarkable (Perera et al., 2022), which coincides with the conclusion of this study. Analyzing from the underlying physiological logic, this intensity range is just like a “golden key.” On the one hand, it provides the muscles with moderate and continuous load stimulation, avoiding excessive fatigue or insufficient stimulation. On the other hand, combined with the local blood flow restricted environment caused by BFR, it promotes a delicate balance in the internal metabolic stress response of the muscles, activates a series of cellular signaling pathways, and efficiently induces the generation of PAP (Kim et al., 2015), which is then transformed into an outstanding improvement in athletic performance.

Conversely, exhaustive exercise protocols (I^2^ = 94%) showed marked variability, underscoring the lack of consensus in defining “exhaustion” and necessitating standardized criteria for future investigations.

#### 4.1.3 Compressive strength

When delving deeper into the key variable of compression intensity, we ingeniously divided the included studies into two subgroups with distinct characteristics: 50% or less of the arterial occlusion pressure (AOP) and 60% or more of the AOP. However, the research results showed that the two subgroups exhibited different performances. When the compression intensity was precisely controlled within the range of ≤50% AOP, the effect size soared to its peak and had a highly significant statistical significance (*SMD* = 0.77, *P* < 0.01). The results indicated that under the condition of a compression intensity of ≤50% AOP, it was easier to stimulate the generation of PAP, opening up a brand-new possible path for the improvement of sports training effects. The lower compression intensity might be like a highly skilled “regulation master.” On the one hand, it can skillfully achieve a moderate blood flow restriction (Pearson and Hussain, 2015). On the other hand, it can also cleverly avoid a series of “adverse chain reactions” that might be triggered by excessive compression, such as tissue damage and nerve feedback inhibition (Neto et al., 2014). The differential effects of low vs. high compression intensities highlight a critical research gap: whether individualized AOP titration (e.g., based on limb morphology or vascular compliance) could further optimize PAP responses remains unexplored.

### 4.2 Impact of BFR training on RPE

Fatigue is a complex physiological phenomenon that involves the interaction of multiple factors (Marcora et al., 2009). This study conducted an in-depth analysis of the impact of BFR training on the rating of perceived exertion (RPE). The results suggest that BFRT can largely influence RPE. During the BFR training process, due to the restriction of limb blood circulation, the metabolites (such as hydrogen ions, inorganic phosphates, *etc.*) produced by muscle contractions cannot be removed as quickly as in the state of normal blood circulation. These metabolites will accumulate in large quantities in the local area of the muscles, thus leading to fatigue (Davis et al., 2024). BFR training will stimulate proprioceptors such as muscle spindles and Golgi tendon organs in the muscles (Ilett et al., 2019). Muscle spindles can sense the changes in muscle length and the speed of stretching, and BFR will increase their discharge frequency. This increased neural impulse will be transmitted to the brain through the spinal cord (Albert et al., 2006). Although the enhanced neuromuscular activation is helpful for improving athletic performance, the continuous high-intensity neural impulse feedback will make the brain perceive a greater load (Noakes, 2012). The brain will interpret this neural feedback as a fatigue signal, thereby increasing the RPE value. In addition, during BFR training, since more attention needs to be paid to the physical sensations (such as the sense of pressure, muscle tension, *etc.*) during the training process, an individual’s attention will be more concentrated on the sensations related to fatigue. This focus of attention will amplify the perception of fatigue (Fahs et al., 2015). Compared with normal training, individuals may be more likely to detect the body’s fatigue signals, thus increasing the RPE value.

On the other hand, the high heterogeneity (*I* ^2^ = 85% > 50%, *p* < 0.01 in the *Q* test) presented in the studies cannot be ignored. This indicates that there are significant differences among different studies, and the sources may be multifaceted. There are differences in the specific implementation details of BFR training among various studies, such as the pressure magnitude and duration of blood flow restriction, the intensity, mode and training cycle of exercise training.

#### 4.2.1 Exercise mode

The impact of BFRT in different exercise modalities on RPE varies. Notably, remarkable results have been achieved in the knee flexion exercise modality. The Standardized Mean Difference (*SMD*) reached 0.65 with *P* = 0.006, clearly revealing that the combination of BFR and knee flexion training can significantly enhance the subjects’ perceived subjective fatigue. On the one hand, the knee flexion movement involves the coordinated operation of multiple muscle groups, including the thigh muscle groups such as the quadriceps femoris and hamstrings. The local blood flow restriction caused by BFR promotes the rapid accumulation of metabolites (such as hydrogen ions, inorganic phosphates, *etc.*) in these muscles during exercise, strongly stimulating the fatigue receptors and afferent nerve fibers within the muscles and efficiently transmitting fatigue signals to the central nervous system, thereby amplifying the perceived subjective fatigue (Wernbom et al., 2013). From an application perspective, this means that when coaches formulate BFR-assisted training plans for athletes, if the plans involve movements related to knee flexion (such as squats, leg curls, *etc.*), they need to be highly vigilant about the potential impact of the rapid accumulation of fatigue on training compliance and subsequent training arrangements.

#### 4.2.2 Exercise intensity

Among numerous exercise intensities, the impact of BFR combined with Mixed-oxygen Training on the subjective fatigue level is the most significant (*SMD* = 1.03, *P* = 0.001). The mixed-oxygen training group combines multiple training methods, posing a comprehensive challenge to the body’s physiological systems. Taking the treadmill exercise to exhaustion as an example, this aerobic exercise process makes the cardiopulmonary function operate at full capacity. Under the effect of BFR, the restriction of limb blood circulation aggravates the hypoxic state of the muscles, causing the rapid conversion from aerobic metabolism to anaerobic metabolism (Proppe et al., 2022). Meanwhile, the 25-min knee flexion exercise, as an important part of strength training, further activates multiple groups of thigh muscles. In the environment of blood flow restriction, the muscles not only have to bear the continuous strength load but also cope with the accumulation of metabolic waste, exacerbating local muscle fatigue (Manimmanakorn et al., 2013). When the exercise intensity is in the range of 40%–70% of the one-repetition maximum (1RM), there is a heterogeneity as high as *I* ^2^ = 93%. The causes behind it are multidimensional and intricate. However, the accumulation of metabolites in this process has a positive effect on stimulating PAP. Future trials should isolate the independent effects of aerobic vs. resistance BFR components to disentangle their contributions to RPE.

#### 4.2.3 Compressive strength

Regarding the impact of BFR training with different compressive strengths on RPE, when the compressive strength reaches ≥60% of the arterial occlusion pressure (AOP), compared with ≤50% AOP, a significantly larger effect size is presented *SMD* = 0.75, *P* = 0.05). Firstly, in the face of higher blood flow restriction, the obstruction to the clearance of metabolites within the muscles becomes more obvious. Metabolites such as hydrogen ions and inorganic phosphates accumulate rapidly (Manimmanakorn et al., 2013). This not only reduces the efficiency of muscle contraction but also stimulates the type III and type IV afferent nerve fibers in the muscles. These nerve fibers are highly sensitive to metabolic changes and they accelerate the transmission of fatigue signals to the central nervous system, causing the subjective fatigue of the subjects to soar (Izquierdo et al., 2009). The accumulation of inorganic phosphates also changes the contractile characteristics of the muscles. Although it helps to some extent in stimulating the post-activation potentiation (PAP) (Yasuda et al., 2014), at the same time, it greatly increases the perception of muscle fatigue. On the other hand, the blood flow restriction under high compressive strength has a huge impact on the oxygen supply to the muscles. The severe shortage of oxygen supply prompts the muscles to rely more on the anaerobic metabolic pathway for energy supply, and the rate of lactic acid production by anaerobic metabolism increases significantly (Yue et al., 2023). The accumulation of lactic acid not only causes muscle soreness but also activates fatigue-related neural feedback through multiple signaling pathways, enabling the brain to receive and interpret stronger fatigue signals, thereby significantly increasing the subjective fatigue level of the subjects (Ishii and Nishida, 2013). The tension between PAP enhancement and RPE elevation at varying pressures underscores the need for individualized BFR prescriptions that balance performance gains with tolerability.

### 4.3 Study limitations

This study has several limitations. On the one hand, the included studies showed a high degree of heterogeneity. Substantial heterogeneity was observed across included studies, primarily attributed to variations in BFR training protocols, participant characteristics, and measurement methods/timing of PAP and RPE outcomes. Notably, gender distribution was incompletely reported in several studies, potentially compromising the accuracy and reliability of subgroup analyses. In terms of testing methods, when using jumping exercises to evaluate the effect of BFR training, details such as the degree of standardization of preparatory movements before jumping, the force generation techniques of participants, and the accuracy and calibration of testing equipment varied among different studies, lacking a unified comparison benchmark. On the other hand, due to the ethical requirements for human experiments, although the subjects signed the informed consent forms, the 31 articles did not adopt the blinding method, which might introduce subjective biases. However, the author believes that this will not have a subversive impact on the results of this study.

## 5 Conclusion

Blood flow restriction (BFR) training can induce the generation of post-activation potentiation (PAP). BFR exercises with an intensity of 40%–70% of the one-repetition maximum (1RM) and a compressive strength of ≤50% of the arterial occlusion pressure (AOP) are more likely to stimulate PAP. Meanwhile, BFR training can significantly affect the rating of perceived exertion (RPE). BFR training combined with mixed-oxygen training and BFR training under a compressive strength of ≥60% AOP result in a stronger perception of fatigue.

## Data Availability

The original contributions presented in the study are included in the article/Supplementary material; further inquiries can be directed to the corresponding author.
